# Global Genomic and Proteomic Analysis Identified Critical Pathways Modulated by Proto-Oncogene PELP1 in TNBC

**DOI:** 10.3390/cancers14040930

**Published:** 2022-02-13

**Authors:** Zexuan Liu, Kristin A. Altwegg, Junhao Liu, Susan T. Weintraub, Yidong Chen, Zhao Lai, Gangadhara R. Sareddy, Suryavathi Viswanadhapalli, Ratna K. Vadlamudi

**Affiliations:** 1 Department of Obstetrics and Gynecology, UT Health San Antonio, San Antonio, TX 78229, USA; zexuanliu1995@outlook.com (Z.L.); altwegg@uthscsa.edu (K.A.A.); liujunhao@csu.edu.cn (J.L.); sareddy@uthscsa.edu (G.R.S.); 2 Department of Oncology, Xiangya Hospital, Central South University, Changsha 410008, China; 3 Mays Cancer Canter, UT Health San Antonio, San Antonio, TX 78229, USA; 4 Department of Biochemistry and Structural Biology, UT Health San Antonio, San Antonio, TX 78229, USA; weintraub@uthscsa.edu; 5 Greehey Children’s Cancer Research Institute, UT Health San Antonio, San Antonio, TX 78229, USA; cheny8@uthscsa.edu (Y.C.); laiz@uthscsa.edu (Z.L.); 6 Department of Population Health Sciences, UT Health San Antonio, San Antonio, TX 78229, USA; 7 Department of Molecular Medicine, UT Health San Antonio, San Antonio, TX 78229, USA; 8 Audie L. Murphy Division, South Texas Veterans Health Care System, San Antonio, TX 78229, USA

**Keywords:** PELP1, DIA-MS, RNA-seq, triple negative breast cancer, ribosome biogenesis

## Abstract

**Simple Summary:**

The proto-oncogene PELP1 is commonly overexpressed in many cancers including triple negative breast cancer (TNBC). In this study, we utilized global proteomic and RNA-seq approaches to elucidate the molecular mechanisms by which PELP1 contributes to the progression of TNBC. Global quantitative proteome analysis revealed that the oncogenic activities of PELP1 involve regulation of the expression of ribosomal proteins, as well as ribosomal regulatory complexes. RNA-seq studies discovered that PELP1 modulates the functions of c-Myc in TNBC, which is a known regulator of ribosomal proteins. Furthermore, TCGA-TNBC data confirmed PELP1 has high expression in TNBC, and this pattern exhibited a positive correlation with c-Myc and regulators of ribosomal proteins. Collectively, our studies suggest that PELP1 contributes to TNBC progression by modulation of ribosome biogenesis pathways.

**Abstract:**

The PELP1 oncogene is commonly overexpressed in many cancers, including triple negative breast cancer (TNBC). However, the mechanisms by which PELP1 contributes to TNBC progression are not well understood. To elucidate these mechanisms, we generated CRISPR-Cas9 mediated PELP1 knockout TNBC cell lines, and alterations in the proteome were examined using global data-independent acquisition mass spectrometry (DIA-MS). Further mechanistic studies utilized shRNA knockdown, Western blotting, and RNA-seq approaches. TCGA data sets were utilized for determining the status of PELP1 in TNBC patient tumors and for examining its correlation with ribosomal proteins. Global DIA-MS studies revealed that 127 proteins are upregulated while 220 proteins are downregulated upon PELP1-KO. Bioinformatic analyses suggested that the oncogenic activities of PELP1 involve regulation of expression of ribosomal proteins and ribosomal complexes. RNA-seq studies further suggested PELP1 modulates the functions of transcription factor c-Myc in TNBC. TCGA data confirmed PELP1 has high expression in TNBC patient tumors, and this high expression pattern correlates with c-Myc, a regulator of ribosomal proteins. Collectively, our global approach studies suggest that PELP1 contributes to TNBC progression by modulation of cell cycle, apoptosis, and ribosome biogenesis pathways.

## 1. Introduction

Triple negative breast cancer (TNBC) is a subtype of breast cancer which lacks the expression of the Estrogen Receptor (ER), Progesterone Receptor (PR), and Human Epidermal Growth Factor Receptor 2 (HER2) [1]. TNBCs are commonly associated with advanced stage disease at presentation, and show a higher propensity for metastasis [2,3]. Currently, TNBC lacks effective targeted therapies, and exhibits a worse overall prognosis [4]. Thus, understanding the mechanisms that drive TNBC progression and the development of effective targeted therapies for women diagnosed with TNBC represents the highest unmet need to improve patient survivorship and quality of life.

Ribosomal biogenesis and translational control play critical roles in the progression of many cancers, including TNBC [5,6]. Several tumor suppressors and proto-oncogenes are known to affect the formation of the mature ribosome or its translation functions [7]. For example, c-Myc-driven tumorigenesis involves regulation of ribosomal genes [8,9]. Similarly, mTOR mediated oncogenic functions involve alterations in ribosomal functions [10]. Further, alterations in the structure of the ribosome [11] and increased ribosomal content in epithelial cells contribute to their enhanced metastatic potential [11]. However, the mechanisms by which oncogenes modulate ribosome biogenesis during TNBC progression have yet to be rigorously investigated.

Proline-, glutamic acid-, and leucine-rich protein 1 (PELP1) was initially identified as a coregulator of the ER [12]. Later studies indicated that PELP1 also functions as a coregulator of other nuclear receptors, including Glucocorticoid Receptor (GR) [13], Androgen Receptor (AR) [14], and transcription factors, such as STAT-3 [15], NF-κB [16], and E2F1 [17]. PELP1/Ki-67 high expression in tumors is an independent prognostic factor for predicting poor survivorship in TNBC patients [18]. Furthermore, PELP1 knockdown reduced the metastatic potential of TNBC cells [19]. In addition, PELP1-targeted therapies enhanced the response of TNBC cells to chemotherapies [17]. These studies suggest PELP1 signaling may play a role in TNBC; however, the molecular mechanisms by which PELP1 contributes to TNBC progression remain elusive.

In this study, using unbiased global quantitative proteomic analyses, we identified that PELP1-mediated oncogenic functions in TNBC involve modulation of ribosome biogenesis. Further, global RNA-seq analyses confirmed PELP1 alters expression of critical regulators of ribosomal genes such as c-Myc. RNA-seq data analyses of TCGA tumors confirmed the positive correlation of PELP1 with ribosomal genes. Collectively, our results indicate that PELP1 regulation of TNBC progression may involve modulation of ribosomal biogenesis.

## 2. Materials and Methods

### 2.1. Cell Culture and Reagents

Human MDA-MB-231 and BT549 cells were purchased from the American-Type Culture Collection (ATCC, Manassas, VA, USA) and cultured in ATCC recommended medium. Cells utilized were free of mycoplasma contamination and identity was confirmed by STR DNA profiling. The PELP1 antibody (A300-180A) was purchased from Bethyl Laboratories (Montgomery, TX, USA). The c-Myc antibody (S1826) was purchased from Clontech (Takara Bio USA, San Jose, CA, USA). The antibody for GAPDH (8884) was purchased from Cell Signaling Technology (Beverly, MA, USA).

### 2.2. Generation of Model Cell Lines

MDA-MB-231 and BT549 cells with stable PELP1 knockdown (KD) were created using validated human specific lentiviral PELP1-shRNA particles (TRCN0000159883, Sigma Burlington, MA, USA). Lentiviral particles expressing non-targeted shRNA (SHC016-1EA, Sigma) were used to generate control cells.

CRISPR/Cas9 mediated gene knockout (KO) cells were generated using CRISPR gRNA construct containing three gRNA sequences (GeneCopoeia, Rockville, MD, USA) and Edit-R Inducible Lentiviral hEF1a-Blast-Cas9 Nuclease Plasmid (Cat.CAS11229, GE Healthcare Dharmacon, Inc., Lafayette, CO, USA). Scrambled sgRNA were used to generate control vector cells (CCPCTR01-LvSG03-B, GeneCopoeia). After transfection, pooled clones were selected under puromycin for two weeks and then screened for PELP1-KO by western blotting. The sequece of three PELP1 gRNAs used in this study are: (1) gtttgctg-caacctcgaacg; (2) ccgggctcatgtgcctattg; and (3) atgtggggccgagcggtttg.

### 2.3. Cell Growth, Colony Formation and Western Blotting

The effects of PELP1-KD and KO on cell growth was measured by MTT assay. TNBC control/vector and PELP1-KD or KO cells were seeded 1000 cells per well in 96-well plates and cell viability was measured every day for four days. For colony formation assays, TNBC model cells were seeded 500 cells per well in 6-well plates and allowed to grow for 14 days. Then, cells were fixed with ice-cold methanol and stained by 0.5% crystal violet solution. Colony numbers were counted by Image J Software [20].

Western blot analyses were performed as described previously [21]. Briefly, TNBC control/vector and PELP1-KD or KO cells were serum starved for 48 h and stimulated with 10% serum for 8 h, then whole-cell lysates were prepared using RIPA buffer containing protease and phosphatase inhibitors (Sigma Chemical Co., St. Louis, MO, USA). Total proteins were mixed with SDS sample buffer and subjected to SDS-PAGE. Blots were developed using the ECL kit (Thermo Fisher Scientific, Waltham, MA, USA).

### 2.4. DIA Mass Spectrometry

**Figure 1:**
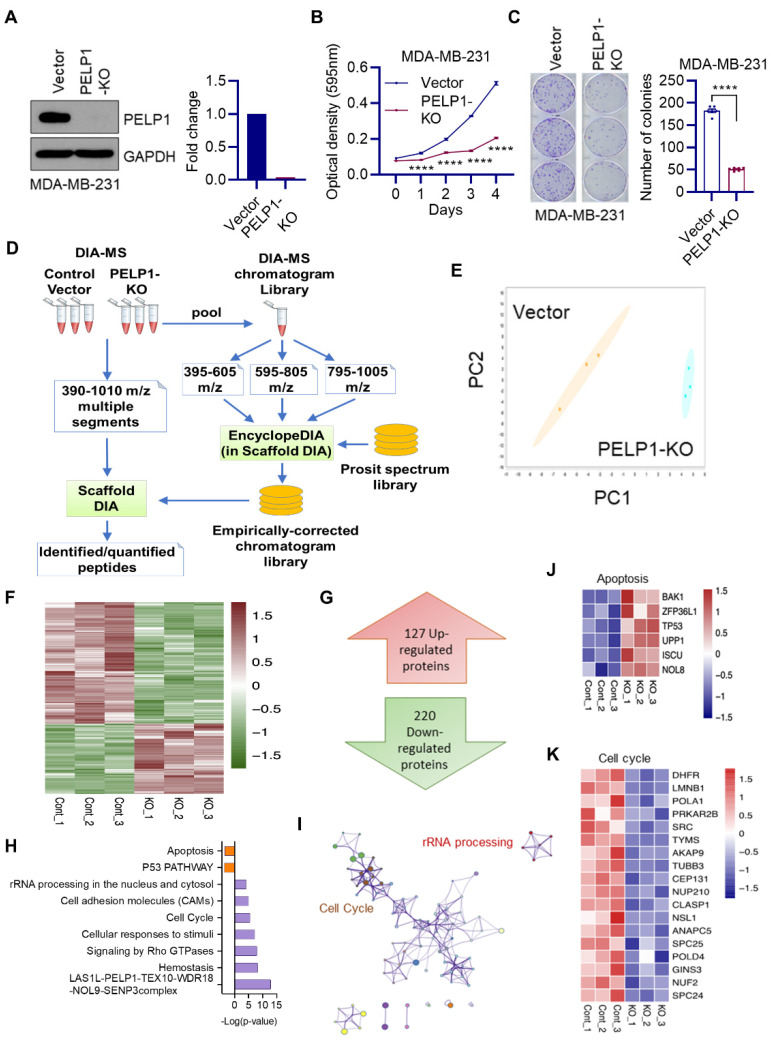
Global DIA-MS identified unique pathways modulated by PELP1 in TNBC. (**A**) Validation of CRISPR/Cas9 mediated PELP1-KO in MDA-MB-231 cells is shown by western blot. Quantitation of PELP1-KO in western blot is shown as right panel. (**B**) Effect of PELP1-KO on cell growth was measured by MTT assay (*n* = 3). (**C**) Effect of PELP1-KO on survival of TNBC cells was determined using colony formation assay (*n* = 3). (**D**) Experimental schema used for DIA-MS analyses is shown. (**E**) PCA of the proteomic data obtained by DIA-MS. (**F**,**G**) Heatmap showing the differentially expressed proteins between vector and PELP1-KO with cut off fold change ≥ 1.5 and *p*-value < 0.05. (**H**) Pathway enrichment analysis of up (red) or down-regulated (purple) proteins upon PELP1-KO. Terms with a *p*-value of < 0.05 were collected. (**I**) Network of representative enriched terms of down-regulated proteins. Terms were hierarchically clustered into a tree based on Kappa-statistical similarities and connected with a similarity kappa score > 0.3. (**J**,**K**) Heatmap confirmed upregulation of apoptosis related proteins (**J**) and downregulation of cell cycle related proteins (**K**) upon PELP1-KO from DIA-MS data. Data are shown as the mean ± SEM. **** *p* < 0.0001.

Total cellular lysates were prepared from MDA-MB-231 vector or PELP1-KO cells, as described in our earlier publication [22], and data-independent acquisition mass spectrometry (DIA-MS) analyses were conducted at the UT Health San Antonio MS Core following the MS Core established protocol. A pool was made of the six samples (biological triplicates from each group), and 2 µg peptide aliquots were analyzed using gas-phase fractionation and 4-*m*/*z* windows (staggered; 30 k resolution for precursor and product ion scans, all in the orbitrap) to create a DIA chromatogram library [23] by searching against a Prosit-generated predicted spectral library [24] based on the UniProt_human_20191022 protein sequence database. Experimental samples were randomized within each replicate for sample preparation and analysis; injections of 2 µg of peptides and a 2-h HPLC gradient were employed. MS data for experimental samples were acquired in the orbitrap using 8-*m*/*z* windows (staggered; 30 k resolution for precursor and product ion scans) and searched against the chromatogram library (Figure 1D). Scaffold DIA (v3.1.0; Proteome Software) was used for DIA-MS data processing. For differential expression analysis, normalized, log2-transformed DIA-MS intensities were used with any missing values discarded. Gene ontology analysis of differentially expressed proteins was conducted focusing on the proteins that exhibited ≥ 1.5-fold change and *p*-value < 0.05 comparing vector and PELP1-KO cells.

### 2.5. RNA Sequencing

Total RNA from control and PELP1-KD MDA-MB-231 cells was isolated using the RNeasy mini kit (Qiagen, Hilden, Germany). RNA-seq was performed using the Genome Sequencing Core (UT Health SA) established protocol and sequencing was done using 50 bp single read sequencing module with Illumina HiSeq 3000 sequencing platform [25]. Differential gene expression analysis was done in DEseq2 [26] and genes with fold change >2 and adjusted *p*-value < 0.05 were categorized as differentially expressed genes. RNA-seq data was deposited in the GEO database with a GEO accession number (GSE191066).

### 2.6. Bioinformatic Analysis

Stand-alone Gene Set Enrichment Analysis (GSEA) [27] was used to perform gene set enrichment. Gene sets used for GSEA were obtained from the Molecular Signatures Database [28]. GO term enrichment analysis was done by Metascape platform [29]. Heatmaps were created by pHeatmap package (https://CRAN.R-project.org/package=pheatmap, accessed on 30 November 2021). Volcano plots were done by ggplot2 package (https://ggplot2.tidyverse.org/, accessed on 30 November 2021). The GeneMANIA prediction server was used to conduct biological network integration for prediction of gene interactions [25]. PELP1 gene expression based on BC subclasss was performed using the UALCAN database [30]. Pearson’s pairwise correlation analyses of TCGA-BRCA dataset was done using bc-GenExMiner 3.0 [31]. Differential PELP1 expression between tumor and normal across diverse cancer types and gene-expression correlation analyses in TNBC was conducted by TIMER2.0 [32]. Overall survival analysis of TNBC patients was done by Kaplan–Meier plotter [33]. PELP1 co-expressed genes were derived from cBioPortal platform [34] and visualized by Cytoscape [35].

### 2.7. Statistical Analysis

The statistical differences were analyzed using student’s t-test with GraphPad Prism 9 software (GraphPad Prism Software, San Diego, CA, USA). All the data represented in bar graphs are shown as mean ± SEM. A value of *p* < 0.05 was considered as statistically significant.

## 3. Results

### 3.1. Global Data-Independent Acquisition Mass Spectrometry (DIA-MS) Identified Unique Pathways Modulated by PELP1

To understand the mechanisms by which PELP1 contributes to the progression of TNBC, we have used PELP1-KO cells. We achieved PELP1-KO in MDA-MB-231 cells using the CRISPR-Cas9 system stably expressing three PELP1 gRNAs along with inducible Cas9. Western blot results confirmed KO of PELP1 (Figure 1A). MTT assays and colony formation assays showed decreased growth (Figure 1B) and survival of PELP1-KO cells, respectively (Figure 1C). We then conducted DIA-MS analysis using whole cell lysates of MDA-MB-231 vector or PELP1-KO cells (Figure 1D). DIA-MS analyses resulted in identification of more than 5000 proteins which were quantified in biological triplicates of the two experimental groups. Unsupervised principal component analysis (PCA) demonstrated the reproducibility and separation between these groups. (Figure 1E). Proteins exhibiting ≥ 1.5-fold change and *p*-value < 0.05 in relative quantity after PELP1-KO compared to vector expressing cells are shown in the heatmaps (Figure 1F).

A total of 127 proteins are upregulated while 220 proteins are downregulated upon PELP1-KO (Figure 1G). We then identified all statistically enriched functional terms of differentially expressed proteins through the Metascape Gene Annotation and Analysis Resource platform. Enriched pathways of the 220 downregulated and 127 upregulated proteins are shown in purple and red, respectively, with the following function annotation databases: CORUM, Reactome Pathway, and KEGG Pathway (Figure 1H). The proteins downregulated by PELP1-KO were enriched in ribosomal biogenesis complex, rRNA processing, and cell cycle while the proteins upregulated upon PELP1-KO belong to apoptosis and p53 pathways (Figure 1H).

To further identify the relationships between the enriched terms of PELP1 positively modulated proteins, a subset of terms was hierarchically clustered into a tree based on Kappa-statistical similarities among their gene memberships. Terms with a similarity kappa score > 0.3 are connected by edges. We identified ribosome biogenesis regulator complexes (LAS1L-PELP1-TEX10-WDR18-NOL-9-SENP) and cell cycle regulator complexes from the cluster network (Figure 1I). Accordingly, analyses of DIA-MS data confirmed upregulation of apoptosis related proteins upon PELP1-KO (Figure 1J) and downregulation of several known proteins involved in cell cycle (Figure 1K).

### 3.2. PELP1 Regulated Proteins in TNBC Cells Play a Critical Role in Ribosome Biogenesis

**Figure 2:**
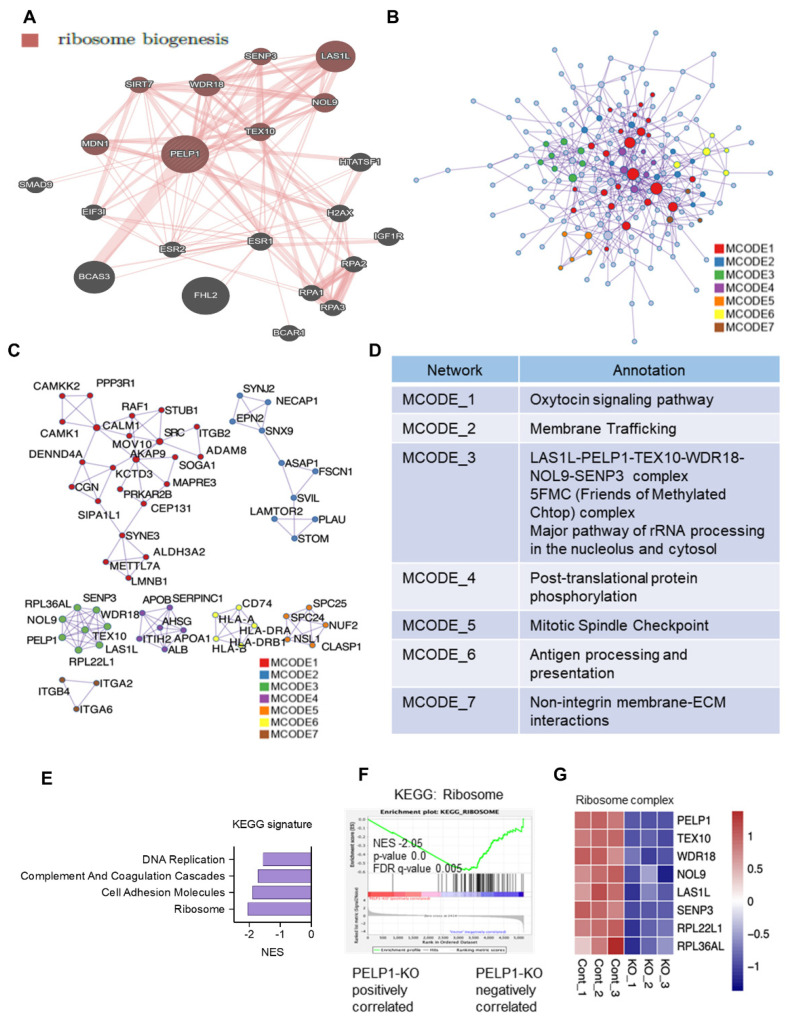
PELP1 regulated proteins in TNBC cells play a critical role in ribosome biogenesis. (**A**) Interactome network of PELP1. Ribosome biogenesis genes are marked red. (**B**) Protein–protein interaction network of 220 downregulated proteins from DIA-MS data. Densely connected proteins were identified by MCODE and named respectively. (**C**) Protein name of each MCODE algorithm identified components are shown. (**D**) Functional annotation of each MCODE component is shown in the tables. (**E**,**F**) GSEA pathway and plot of enriched KEGG gene sets. (**G**) Heatmap confirmed down-regulation of several known proteins involved in ribosomal biogenesis.

Since proteins downregulated upon PELP1-KO are over-represented in ribosomal biogenesis pathways, we examined the network of PELP1 interactions and confirmed that PELP1 has close interactions with several ribosomal regulatory proteins including LAS1L, TEX10, WDR18, and SENP3 (Figure 2A). We then conducted protein–protein interaction enrichment analysis using proteins whose status is altered by PELP1-KO (Figure 2B). These networks include proteins that form physical interactions with at least one other member. For further analyses, Molecular Complex Detection (MCODE) algorithm was then applied to this network to identify densely connected protein components; a functional description has been conducted for each MCODE network component independently. We identified several key regulatory components including the ribosomal biogenesis complex and rRNA processing as the major network components (Figure 2C,D).

To extend the functional analysis to all quantified proteins without threshold, we conducted GSEA using the KEGG gene set. The results showed that several biological pathways were downregulated in the PELP1-KO group including cell adhesion, complement and coagulation, and DNA replication, suggesting these functions are impaired when PELP1 is knocked out in TNBC (Figure 2E). We found specifically that the “ribosome” pathway was the top enriched KEGG pathway, confirming that elimination of PELP1 impairs ribosome biogenesis activity (Figure 2F). Further, analyses of the DIA-MS data also confirmed downregulation of several known proteins involved in ribosomal biogenesis (Figure 2G). Altogether, these results suggest that PELP1 plays an essential role in regulating the expression of proteins involved in ribosome biogenesis.

### 3.3. Global RNA-Seq Analyses Revealed PELP1 Knockdown Impairs Expression of Critical Regulators of Ribosomal Genes Such as c-Myc

**Figure 3:**
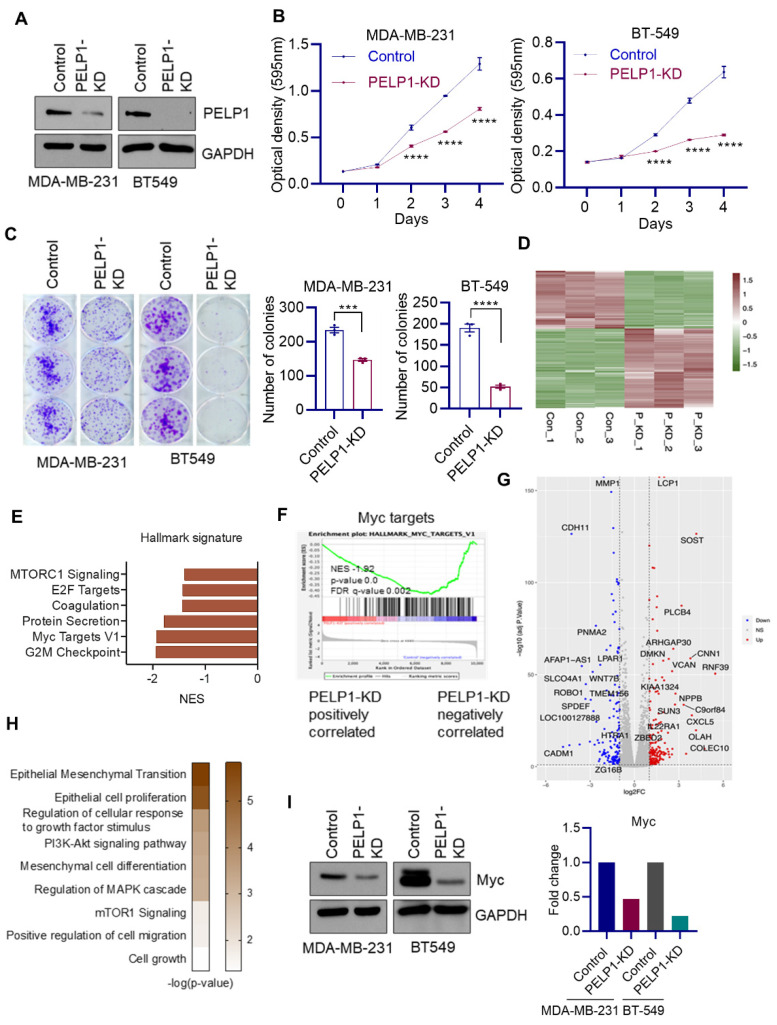
Global RNA-seq analyses revealed PELP1-KDimpairs expression of c-Myc targets. (**A**) Validation of PELP1-KD in two TNBC model cell lines by western blotting. (**B**) Effect of PELP1-KD on TNBC cell growth was measured by MTT assay (*n* = 3). (**C**) Effect of PELP1-KD on survival of TNBC cells was determined using colony formation assay (*n* = 3). (**D**) Heatmap shows differentially expressed genes from RNA-seq data. (**E**) Enrichment of GSEA Hallmark pathways positively correlated with PELP1. (**F**) GSEA plots shows c-Myc target gene signatures were negatively enriched with PELP1-KD regulated genes. (**G**) Volcano plot showing differentially expressed genes in PELP1-KD cells. (**H**) Functional enrichment of genes positively correlated with PELP1. (**I**) TNBC model cells were serum starved for 48 h, stimulated with 10% serum for 8 h and c-Myc expression was determined by western blotting. Quantitation of western blots is shown as right panel. Data are shown as the mean ± SEM of three experiments. *** *p* < 0.001; **** *p* < 0.0001.

To elucidate the mechanisms by which PELP1 alters gene expression in TNBC cells, we established PELP1-KD cells using validated PELP1shRNA. PELP1-KD cells showed 60–70% decrease in PELP1 expression compared to control-shRNA expressing cells (Figure 3A). PELP1-KD TNBC cells showed decreased cell growth (Figure 3B) and cell survival (Figure 3C). Since PELP1 functions as a coregulator of several transcription factors, we reason that global transcriptomic profiling will provide critical information of the mechanisms by which PELP1 regulates TNBC progression. Therefore, we conducted RNA-seq of MDA-MB-231 control and PELP1-KD cells. These studies identified 86 downregulated and 119 upregulated genes upon PELP1-KD with adjusted *p*-value < 0.05; log_2_FC > 1; average log2(RPKM) > 0 (Figure 3D). GSEA hallmark gene set analysis revealed a positive correlation between PELP1-regulated genes with signatures of c-Myc, E2F, G2M, and mTOR (Figure 3E). Importantly, we found that gene sets of c-Myc targets were downregulated in the PELP1-KD group, confirming that deletion of PELP1 decreases c-Myc activity (Figure 3F).

Differential expression profiling between control and PELP1-KD are shown in the volcano plots (Figure 3G). Enrichment analysis confirmed that downregulated genes upon PELP1-KD were involved in EMT, cell growth, migration, PI3K-Akt, and mTOR pathways (Figure 3H), which indicates that PELP1 is positively correlated with these functions in TNBC. We also independently validated that PELP1-KD reduces expression of c-Myc using western blotting (Figure 3I). Collectively, these data suggest that PELP1 plays an integral role in the regulation of a subset of genes regulated by key transcription factors implicated TNBC progression.

### 3.4. PELP1 Expression Is Upregulated and Positively Correlates with c-Myc Status and Ribosomal Regulators in TNBC Samples

**Figure 4:**
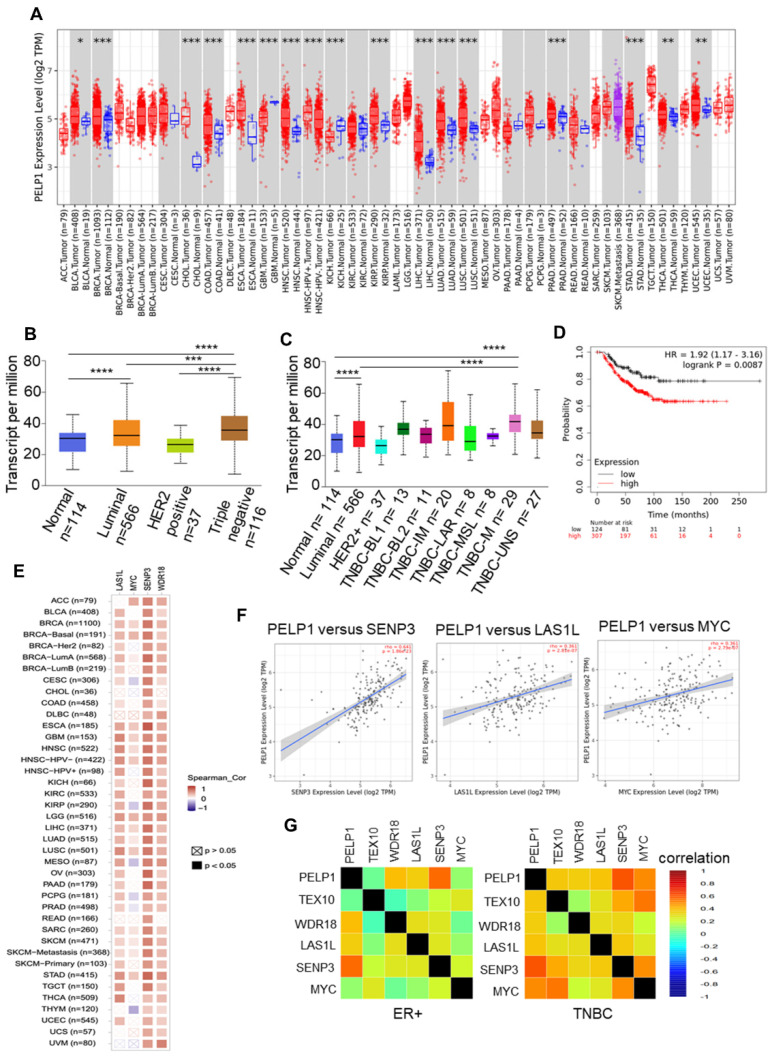
PELP1 expression is upregulated and positively correlates with c-Myc and ribosomal regulators in TNBC. (**A**) PELP1 expression between tumor and normal samples across multiple cancer types. (**B**,**C**) Graph derived from TCGA-BRCA data available in UACLAN database. The box charts depict the relative expression of PELP1 in BC subtypes (**B**) and TNBC molecular subclasses (**C**). (**D**) Overall survival in BC patients was determined by Kaplan–Meier analysis and significant differences were determined by the log-rank test. (**E**,**F**) Gene correlation analysis of PELP1 and indicated genes in various cancer types (**E**) and BRCA-basal (**F**). Correlation heatmap shows the purity-adjusted partial spearman’s rho value representing the degree of correlation. (**G**) Pearson’s pairwise correlation plot of PELP1 and indicated genes in ER+ and TNBC. The color represents the degree of correlation. * *p*<0.05; ** *p*<0.001; *** *p* < 0.001; **** *p* < 0.0001.

Using TCGA datasets that contain gene expression profiles of tumor tissues, we discovered that PELP1 is highly expressed across diverse cancer types (Figure 4A). In breast cancer subtypes, expression of PELP1 is significantly higher in TNBC compared to luminal and HER2-positive BC (Figure 4B). Further analyses of PELP1 expression among the TNBC molecular subclasses showed that PELP1 is highly expressed in TNBC-Mesenchymal (M) and TNBC-Immunomodulatory (IM) subtypes (Figure 4C). Survival analyses revealed a positive correlation between high PELP1 expression and poor survival in TNBC patients (Figure 4D). Since PELP1-KO affected c-Myc target genes and a number of proteins involved in ribosomal biogenesis, such as SENP3, LAS1L, and WDR18, we examined the co-expression of these proteins with PELP1 in tumors.

The results revealed a positive correlation in various cancer types including BRCA-basal (Figure 4E). In particular, PELP1 expression is highly correlated with SENP3, LAS1L, and c-Myc in BRCA-basal tumors (Figure 4F). From additional analyses of the correlation in BC subtypes, we found that the positive correlation of PELP1 and c-Myc only occurs in TNBC, and not in ER+ subtypes of BC. This suggests a unique function of PELP1 in TNBC (Figure 4G). Altogether, it can be concluded from our data that PELP1 is highly expressed in multiple cancer types (versus their corresponding normal)—especially in TNBC. The correlative relationship between PELP1 with c-Myc and ribosomal regulators is indicative of the critical role it plays in modulating TNBC progression via regulation of c-Myc and ribosome biogenesis.

### 3.5. TCGA-TNBC Cohort RNA-Seq Data Revealed PELP1 Is Involved in Regulation of c-Myc and Ribosome Biogenesis

**Figure 5:**
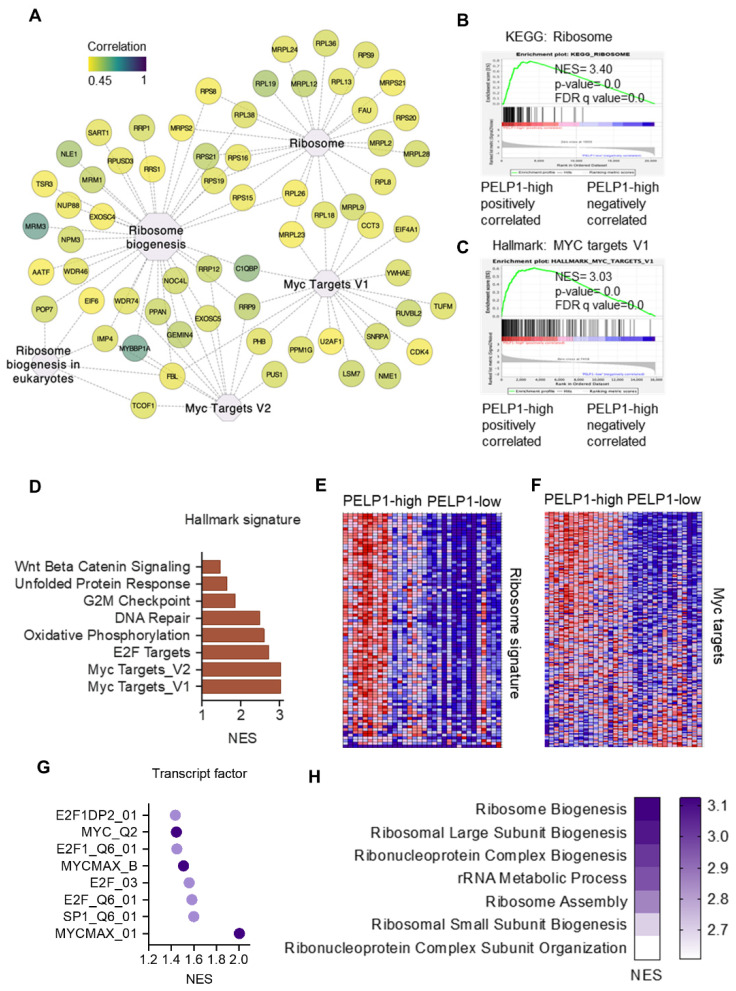
Analyses of gene expression in RNA-seq datasets of TNBC patients revealed PELP1 is involved in regulation of c-Myc and ribosome biogenesis. (**A**) Functional enrichment of top 300 PELP1 positively co-expressed genes derived from TCGA-BRCA-basal patient cohort. Center nodes show enriched pathway and node color represents the correlation degree between indicated genes with PELP1. Data was visualized by Cytoscape. (**B**,**C**) GSEA plots shows ribosome (**B**) and c-Myc (**C**) gene signatures enriched in the PELP1-high group. (**D**) Top enrichment of Hallmark gene signature in PELP1-high group. (**E**,**F**) Model heatmap shows ribosome and c-Myc signature expression between PELP1-high and PELP1-low groups. (**G**,**H**) Enrichment of transcription factor (**G**) and GO Biological function (**H**) from GSEA.

To better understand PELP1 functions in TNBC, we selected the top 300 PELP1 positively co-expressed genes using the TCGA-TNBC cohort. Pathway analyses using KEGG, Hallmark, and Gene Ontology (GO) Biological functions revealed that enrichment includes ribosome biogenesis and c-Myc targets, suggesting that PELP1 is closely related with these biological functions in TNBC (Figure 5A).

To extend the transcriptional analysis to patient’s data, we then analyzed RNA-seq data derived from 161 TNBC tumor samples in the Cancer Genome Atlas Breast Invasive Carcinoma (TCGA-BRCA) cohort. We ranked these tumors by relative PELP1 expression in the top 10th percentile (PELP1-high, *n* = 16) and the bottom 10th percentile (PELP1-low, *n* = 16). GSEA was then used to identify pathways positively correlated with higher PELP1 expression level. The results showed that ribosome and c-Myc target gene signatures were enriched in PELP1-high tumor samples in comparison with PELP1-low samples (Figure 5B,C). Top enriched Hallmark gene signature confirmed similar pathways identified in our genomic and proteomic analysis including pathways modulated by c-Myc, E2F, and G2M (Figure 5D). Further analyses of TCGA data sets confirmed correlation of PELP1 high expression with ribosome and c-Myc targets (Figure 5E,F). Transcription factor motif analyses confirmed enrichment of E2F1, c-Myc, and SP1 in the promoters of PELP1 regulated genes (Figure 5G). Pathway analysis confirmed the correlation of PELP1 regulated gene signatures with ribosome biogenesis and other ribosomal related pathways (Figure 5H). Collectively, these results suggest that PELP1 expression is positively correlated with c-Myc and ribosome biogenesis gene signatures in TNBC.

## 4. Discussion

The oncogene PELP1 is highly expressed in breast tumors. PELP1 expression serves a prognostic predictor of shorter BC-specific survival and disease-free interval [36]. However, the molecular mechanism by which PELP1 promotes TNBC progression remain inadequately studied. In this study, using global unbiased DIA-MS analyses of control vector and PELP1-KO cells, we found that PELP1-KO alters the levels of ribosomal proteins. Further, global RNA-seq analyses indicated that PELP1-mediated signaling in TNBC involves modulation of c-Myc and mTOR signaling which regulate ribosomal proteins and E2F which regulates cell cycle proteins.

Earlier published studies suggested that PELP1 plays an essential role in ribosomal biogenesis. Specifically, the PELP1–TEX10–WDR18 complex has been identified as a regulator of ribosome biogenesis. Moreover, the SUMO-controlled distribution of PELP1 coordinates the rate of ribosome formation [37]. In addition, PELP1 localizes to the nucleolus and plays an essential role in the efficient synthesis of 28S rRNA [38]. PELP1 is implicated as a regulatory point for mammalian 60S maturation through ordered recruitment and release of AAA ATPase MDN1 [39]. Our data using global DIA-MS studies suggest that PELP1 also plays a role in the regulation of expression of several ribosomal proteins. Further, our studies suggest that PELP1 is required to maintain the status of regulatory complexes such as LAS1L–PELP1–TEX10–WDR18–NOL-9–SENP3.

PELP1 high expression in tumors is a known independent prognostic factor for predicting poor survivorship in patients diagnosed with TNBC [18]. PELP1 signaling plays a critical role in TNBC metastases [19,40], and metastatic tumors exhibit increased PELP1 expression compared to node-negative specimens [41]. Further, PELP1 signaling has a critical role in TNBC cell migration and modulates expression of genes involved in EMT/metastasis [19]. It is also known that PELP1 can interact with GR to activate Breast Tumor Kinase (BRK) expression in TNBC [13]. Our data suggest that PELP1 regulation of ribosomal proteins is concomitant with regulation of TNBC progression. We speculate that PELP1 contributes to TNBC progression by modulating both expression of ribosomal proteins and ribosome biogenesis.

PELP1 promotes tumorigenesis by accelerating cell cycle progression, and PELP1 is a substrate of CDK4 kinase [42]. Depletion of PELP1 induced cell cycle arrest accompanied by autophagy [43]. PELP1 localizes to the nucleolar compartment in a cell cycle stage-dependent manner and facilitates optimal ribosomal RNA synthesis [44]. PELP1 has also been shown to increase the expression of E2F1 target genes [17]. Our RNA-seq results corroborate earlier published studies and further suggest that PELP1 can modulate the functions of several transcription factors including E2F1 in TNBC.

Oncogenic PELP1 signaling is implicated in the progression of several cancers, including breast [12], endometrial [45], ovarian [46], salivary [47], prostate [14], lung [48], pancreas [49], and colon [50]. Our results using bioinformatic analyses of TNBC gene expression data sets identified that ribosome and c-Myc-target gene signatures were enriched in PELP1-high tumor samples compared to PELP1-low tumor samples. Further, PELP1 associated genes are enriched with E2F1, SP1, and c-Myc regulatory sites in their promoters and exhibited a positive correlation with PELP1 expression. Based on these results, we speculate that PELP1 expression, along with its associated/regulated ribosomal genes, may serve as a better prognostic marker of survival in TNBC patients.

## 5. Conclusions

The results of global proteomic analyses using DIA-MS revealed that the oncogenic activities of PELP1 involve regulation of expression of ribosomal proteins and ribosomal complexes. Further, RNA-seq studies suggested that PELP1 modulates the functional targets of several transcription factors, such as c-Myc and E2F1, in TNBC. Collectively, our studies indicate that PELP1 contributes to TNBC progression by modulation of cell cycle, apoptosis, and ribosomal biogenesis pathways. Therefore, PELP1 status, along with ribosomal proteins, can serve as prognostic factor for predicting TNBC patient survival.

## Data Availability

All data generated for this study are included within this article. RNA-seq data has been deposited in the GEO database under a GEO accession number (GSE191066).

## Supplementary Materials

The following supporting information can be downloaded at: https://www.mdpi.com/2072-6694/14/4/930/s1, Figure S1: Original western blots for Figure 1A. Cropped sections used as figures in the manuscript are marked as a box. Figure S2: (A) Original western blots for Figure 3A. Cropped sections used as figures in the manuscript are marked as a box. (B) Quantitation of western blot data. Figure S3: Original western blots for Figure 3I. Cropped sections used as figures in the manuscript are marked as a box.
