# Supplementary material for: Global Genomic and Proteomic Analysis Identified Critical Pathways Modulated by Proto-Oncogene PELP1 in TNBC
Source: Cancers (Basel). 2022 Feb 13;14(4):930. doi: 10.3390/cancers14040930 (PMC8924758; doi:10.3390/cancers14040930)
Supplement: Supplementary file 1 [file cancers-14-00930-s001.zip › cancers-1555130-SI.pdf]

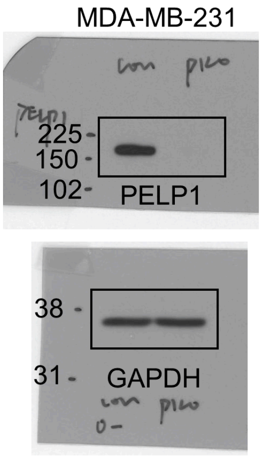

Figure S1. Original western blots for Figure 1A. Cropped sections used as figures in the manuscript are marked as a box.

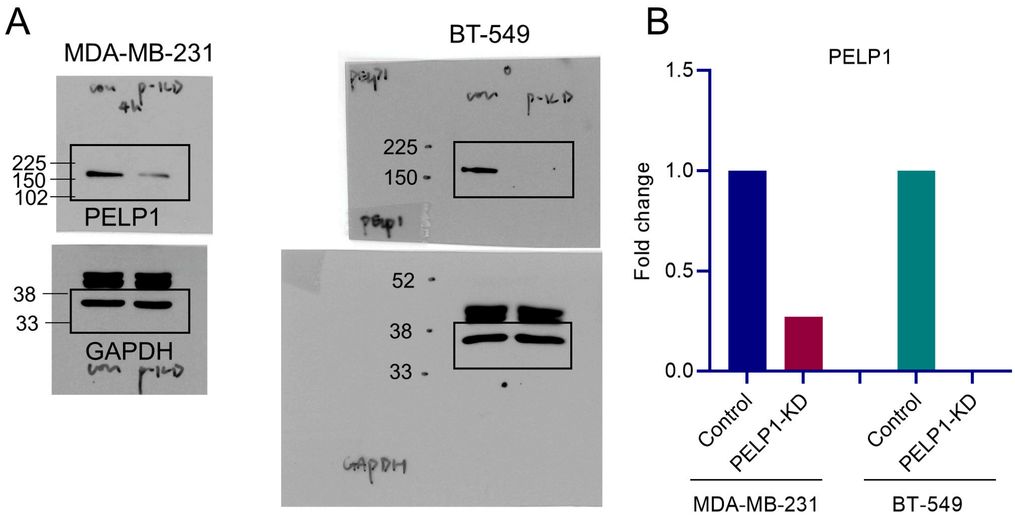

Figure S2. (**A**) Original western blots for Figure 3A. Cropped sections used as figures in the manuscript are marked as a box. (**B**) Quantitation of western blot data.

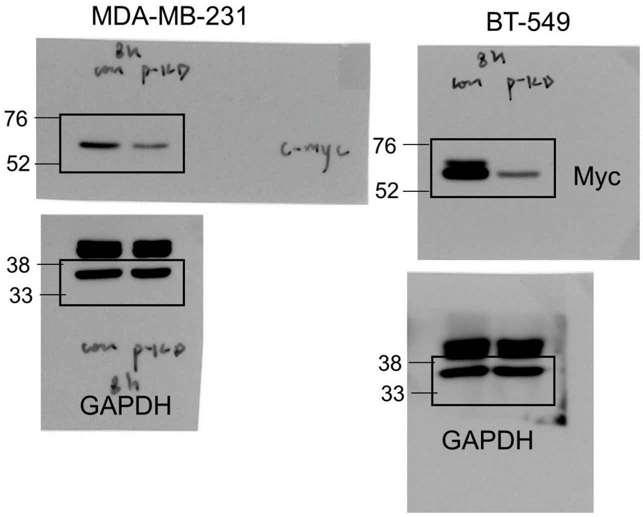

Figure S3. Original western blots for Figure 3I. Cropped sections used as figures in the manuscript are marked as a box.
